# Accurate Surface and Finite-Temperature Bulk Properties of Lithium Metal at Large Scales Using Machine Learning Interaction Potentials

**DOI:** 10.1021/acsomega.3c10014

**Published:** 2024-02-21

**Authors:** Mgcini Keith Phuthi, Archie Mingze Yao, Simon Batzner, Albert Musaelian, Pinwen Guan, Boris Kozinsky, Ekin Dogus Cubuk, Venkatasubramanian Viswanathan

**Affiliations:** † Department of Mechanical Engineering, Carnegie Mellon University, Pittsburgh 15213, Pennsylvania, United States; ‡ School of Engineering and Applied Science, Harvard University, Cambridge 02138, Massachusetts, United States; § Google DeepMind, Kings Cross, London N1C4AG, U.K.

## Abstract

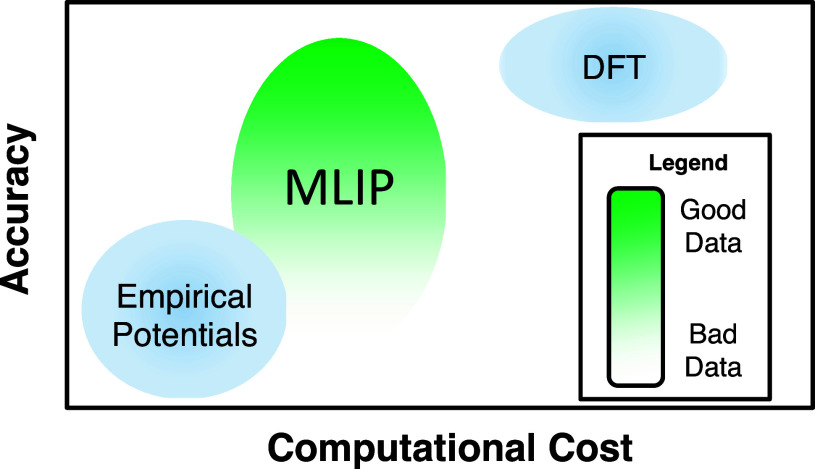

The properties of lithium metal are key parameters in the design of lithium-ion and lithium-metal batteries. They are difficult to probe experimentally due to the high reactivity and low melting point of lithium as well as the microscopic scales at which lithium exists in batteries where it is found to have enhanced strength, with implications for dendrite suppression strategies. Computationally, there is a lack of empirical potentials that are consistently quantitatively accurate across all properties, and ab initio calculations are too costly. In this work, we train a machine learning interaction potential on density functional theory (DFT) data to state-of-the-art accuracy in reproducing experimental and ab initio results across a wide range of simulations at large length and time scales. We accurately predict thermodynamic properties, phonon spectra, temperature dependence of elastic constants, and various surface properties inaccessible using DFT. We establish that there exists a weak Bell–Evans–Polanyi relation correlating the self-adsorption energy and the minimum surface diffusion barrier for high Miller index facets.

## 1 Introduction

Lithium-metal batteries (LMBs) provide a promising pathway to achieving high-capacity energy storage devices. However, realizing practical LMBs has been limited by morphological instabilities, primarily related to dendrite formation and thus safety issues.^1−3^ A number of approaches have been proposed to address the issue of dendrite formation. One approach is suppressing instability through the introduction of a solid electrolyte in contact with lithium. Monroe and Newman proposed using a solid polymer electrolyte with a shear modulus larger than that of lithium,^4^ which was extended by Ahmad and Viswanathan showing that an alternate approach could be to use a lithium-dense solid electrolyte whose modulus is smaller than that of lithium.^5^ A second approach increases morphological stability through rapid surface diffusion, quantified by surface diffusion barriers.^6,7^ All of these approaches critically hinge on accurate determination of the properties of lithium metal. The first approach requires knowing the mechanical properties of lithium at operating temperatures, while the second approach requires a detailed understanding of surface diffusion barriers across different Miller index facets formed during morphological instability. Determining these properties experimentally is challenging due to the high reactivity of lithium,^8^ and thus, computational methods provide a practical approach.

In principle, many material properties can be calculated using high fidelity atomistic methods such as ab initio molecular dynamics (AIMD). In practice, however, the computational cost of these methods is often prohibitive due to poor scaling with the number of electrons [∼O(*N*^3^)] and the long simulation time.^9^ Instead, empirical potentials are commonly used for molecular dynamics (MD) to simulate for the necessary time and length scales, often with a considerable loss of accuracy. In the past decade, a number of machine learning interaction potentials (MLIPs) have been developed, which demonstrate remarkable accuracy in reproducing ab initio results compared to empirical potentials if trained with a sufficiently sampled data set.^10−12^

MLIPs have been used to study supercritical phenomena in hydrogen^13^ and defects in various metals,^14^ have been benchmarked for transition metals,^15^ and have many other applications. Commonly among these examples, the MLIPs are not trained for large regions of the phase space to produce a single, accurate potential. For lithium, the SNAP potential^16^ has been developed for purposes of benchmarking MLIPs and is therefore limited in its applicability, as we show in this work. Jiao et al. generated a deep potential (DP)^17^ and simulated the self-deposition of Li and the different morphologies that could arise in deposition processes.^18^ Their potential, however, was not accurate in predicting stresses and elastic constants, which we improve upon in our own DP in the Supporting Information.

In this work, we generate data to train an MLIP for pure lithium metal based on the neural equivariant interatomic potential (NequIP) architecture.^11^ We also developed a DP and present comparisons in the Supporting Information. The NequIP reproduces density functional theory (DFT) and experimental results remarkably well over a wide range of structures including, bulk, surfaces, defects and liquids, all in one potential, consistently outperforming empirical potentials and existing MLIPs. We therefore more accurately calculate elastic and surface properties important to the design of LMBs and discuss the implications of our results.

## 2 Methods

In this section, we describe the model architectures, data generation, and simulation details. Further details can be found in the Supporting Information.

### 2.1 Machine Learning Interaction Potentials

MLIPs are parametric models that can be trained on a set of atomic configurations given in terms of coordinates {**R**}, including any periodic unit cells and labeled with the corresponding energies, forces, and stresses for that configuration from a high fidelity source such as DFT.

Typically, the total energy of an atomic configuration is modeled as a sum of atomic energies (ε_*i*_) that are dependent on the local environments of the central atom *i*, i.e.,
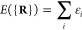


The *i*-th atom contributes an energy (ε_*i*_) that is learned from the geometry centered on atom *i*. The forces on each atom and the virial stresses can then be calculated as the derivatives of the total energy with respect to the atomic positions and the cell dimensions, respectively, using automatic differentiation. It is essential that the predicted energies, forces, and stress are appropriately equivariant with respect to translations, rotations, and permutations of atoms of the same species. The key difference between different potentials is how they implement this equivariance.

The NequIP is an *E*(3)-equivariant message passing interatomic potential that was shown to demonstrate state-of-the-art accuracy, sample efficiency, and transferability on a variety of materials systems at the time of writing.^11,19,20^ While conventional interatomic potentials operate on invariant descriptors of the materials systems, such as distances and angles,^21,22^ the NequIP directly operates on relative interatomic positions  represented as a graph and leverages latent features composed of not only scalar but also vector and higher-order tensor features.

### 2.2 DFT Data

**Figure 1:**
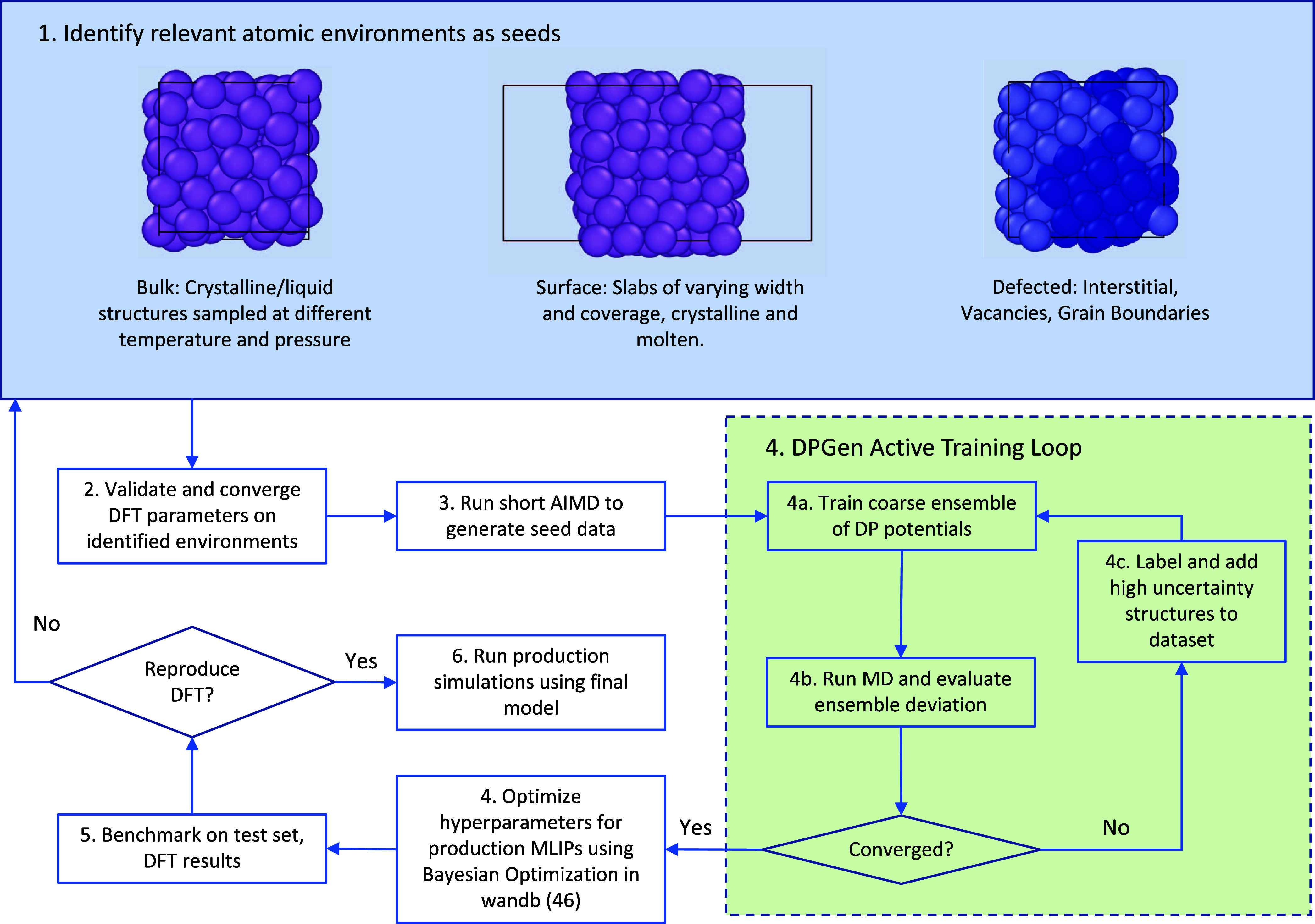
Schematic showing the process by which data were generated and potentials were validated.

A schematic of the procedure used to generate the data is shown in Figure 1. All data used to train the MLIPs were generated using DFT with the same parameters across the entire data set. The parameters chosen were such that the Brillouin zone sampling density and plane wave and density cutoffs were converged to <1 meV/atom to ensure consistency and give a concrete estimate of statistical noise in the data. DFT calculations were performed using Quantum Espresso^23^ within the generalized gradient approximation using the Perdew–Burke–Ernzerhof exchange correlation functional^24^ and the projector augmented wave approach^25^ with a plane wave cutoff energy of 1360 eV. We used the pseudopotential Li.pbe-s-kjpaw_psl.1.0.0.UPF from http://www.quantum-espresso.org. A uniform Brillouin zone spacing of 0.02 Å^–1^ with a Monkhorst–Pack^26^ sampling procedure was used. To help with convergence of the Fermi surface, Methfessel–Paxton^27^ smearing using a smearing width of 0.27 eV was chosen.

The data used to train the potentials were sampled using an active learning approach as implemented in the DPGen package.^28^ A variety of seed structures including bulk crystals, monovacant, monointerstitial, and clean surfaces, and surfaces with varying coverage were generated. The seed structures were used as starting structures for isobaric, isothermal (NPT) MD simulations using a Nosé–Hoover style thermostat and barostat equations of motion by Shinoda et al.^29^ The NPT simulations for generating new structures were performed using an ensemble of coarsely trained DP models in each active learning step using the large-scale atomic/molecular massively parallel simulator (LAMMPS). The MD simulations were performed sequentially on a grid with temperatures of 50, 300, 450, and 900 K, i.e., double the melting temperature, and pressures of 0, 0.1, 1, and 10 GPa. With each active learning step in the sequence, data from the previous steps were incorporated into subsequent steps to improve accuracy and flag new structures with high uncertainty for labeling with DFT. The final data set used to train production potentials was split into 4548 structures in the training set and 505 configurations in the test set.

## 3 Results

We first demonstrate the accuracy of NequIP MLIPs by comparing the predictions directly to results from DFT, other potentials in the literature, and the experimental results. Finally, we predict key properties in the design of LMBs that have been computationally inaccessible by using highly accurate and relatively fast NequIP potentials. We show results for NequIP MLIPs trained on the same data but with model parameters having different float point precisions. The potentials are mostly equivalent, except for the fact that NequIP64, with double precision, is slightly more accurate when calculating energy differences and NequIP32, with single precision, is slightly faster and more memory efficient and is thus used for MD. The subtleties in the differences are explained in the Supporting Information.

### 3.1 Benchmarks against Density Functional Theory

For comparison, we train a DP, a popular architecture, used in the active learning loop described in the Methods section for comparison. Where possible, we also compare our results with the MEAM empirical potential developed by Kim et al.^30^ and the SNAP MLIP developed by Zuo et al.^16^ in the text or the Supporting Information. The MEAM potential has been used to predict the thermal behavior in lithium-metal electrodes^31^ and the lifetime of glassy lithium nuclei under fast charging conditions.^32^ The ReaxFF family of potentials is also used to study lithium in electrode materials, but none have been designed specifically for pure lithium.^33^ It is worth noting that the differences in predictions can be attributed not only to the quality of the fit but also to the quality of the training data sets. The potentials therefore need to be compared with experiments to be assessed rigorously.

Predictions for a number of different properties accessible by using standard DFT methods are shown in Table 1 to benchmark the MLIPs. Details on how the calculations were performed are given in the Supporting Information.

**Table 1:** Various Properties of BCC Lithium Predicted Using The MLIPs and Compared to DFT Results in the Literature, DFT Results in This Work, and the Existing MEAM and SNAP Potentials^a^

property	DFT (other work)	DFT (this work)	DeepMD	NequIP32	NequIP64	SNAP	MEAM
energy RMSE (meV/atom)			3	1	1		
force RMSE (meV/Å)			20	12	12		
stress RMSE (GPa)			0.22	0.06	0.06		
evaluation time (s)		10^4^	10^–3^	10^–1^	10^–1^	10^–2^	10^–4^
lattice constant (Å)	3.427^16^	3.434	3.434 [0.0]	3.431 [−0.1]	3.429 [−0.1]	3.494 [1.7]	3.506 [2.1]
*E*_v_ (eV/atom)	0.62^16^	0.525	0.518 [−1.4]	0.567 [7.9]	0.520 [−0.9]	0.486 [−7.4]	0.378 [−27.9]
bulk modulus (GPa)	14^16^	13.7	13.7 [0.0]	14.0 [2.2]	14.0 [2.3]	10.5 [−23.7]	12.9 [−5.7]
*C*_11_ (GPa)	15^16^	14.8	14.2 [−4.3]	14.9 [0.4]	14.7 [−0.4]	18.4 [24.0]	17.9 [21.2]
*C*_12_ (GPa)	13^16^	13.1	13.5 [2.4]	13.6 [3.2]	13.6 [3.8]	6.5 [−50.5]	10.4 [−20.9]
*C*_44_ (GPa)	11^16^	10.4	13.2 [26.5]	10.9 [4.7]	10.9 [4.7]	10.0 [−3.7]	12.7 [22.3]
anisotropy	11^16^	12.6	37.2 [196.1]	16.8 [33.3]	19.6 [56.1]	1.7 [−86.5]	3.4 [−73.1]
(100) surf. energy (eV/Å^2^)	0.029^34^	0.029	0.029 [0.5]	0.029 [−0.8]	0.029 [−0.7]	0.027 [−7.1]	0.024 [−15.9]
(110) surf. energy (eV/Å^2^)	0.031^34^	0.031	0.031 [0.0]	0.031 [−0.6]	0.031 [−0.1]	0.028 [−9.4]	0.024 [−21.9]
(111) surf. energy (eV/Å^2^)	0.034^34^	0.033	0.034 [3.0]	0.033 [0.9]	0.033 [0.2]	0.030 [−8.6]	0.028 [−14.6]
HCP Δ*E* (meV/atom)	0^35^	0	–2	0	0	–6	0
FCC Δ*E* (meV/atom)	0^35^	0	0	0	0	0	0
BCC Δ*E* (meV/atom)	2^35^	2	1	1	1	–2	0

a Percentage errors relative to the DFT prediction are shown in square brackets. Except for the anisotropy, all the errors are within 5% for the NequIP64 potential. The NequIP32 has a large vacancy formation energy error due to the lower precision used to calculate small energy differences. The SNAP and MEAM are likely to produce different results as they were trained on different data and parameterized differently than in this work.

The root mean square errors (RMSEs) shown in Table 1 demonstrate very good accuracy on the order of ∼1 meV/atom for energies, comparable to the fluctuations in the energy with converged *k*-points and typically chosen DFT convergence criteria.^36^ The forces and stresses are also well converged to ∼10 meV/Å and ∼1 meV/Å^3^, respectively, such that an atomic/cell parameter displacement of ∼0.01 Å gives an energy difference of ∼1 meV/atom. It is worth noting that NequIP potentials perform an order of magnitude better in terms of the stress error (0.06 eV/Å^3^) than the DP (0.22 eV/Å^3^) and are slightly better than the DP in most other metrics.

The RMSE is a good metric for benchmarking MLIP architectures against each other but does not imply an accurate potential in predicting experimental properties since it strongly depends on an inevitably biased test set. We therefore calculate a number of other properties to benchmark the MLIPs that reflect the quality of the labeling and distribution of data in the training set. Table 1 shows properties calculated, which are in excellent agreement, with most being within 5% of the DFT predictions for the DP and NequIP potential. An exception is the anisotropy, which is derived from the elastic constants as 2*C*_44_/(*C*_11_–*C*_12_) and hence propagates the error from the elastic constants.

It is worth noting how much faster the MLIPs are compared to DFT. The table shows a typical order of magnitude run time for a 128-atom unit cell. The NequIP models are ∼5 orders of magnitude faster than DFT with little loss in accuracy with respect to DFT, making them the ideal choice. In general, the time cost of each evaluation depends on the number of atoms and hardware, but the linear scaling in the cost of MLIPs is significantly more favorable than that of DFT.

A number of other benchmarks are included in the Supporting Information to demonstrate the reliability of the NequIP potentials over the other potentials considered in this work. These benchmarks include parity plots, energy–volume curves, surface energies, and Wulff constructions.

### 3.2 Temperature Dependence of Elastic Properties of Lithium

**Figure 2:**
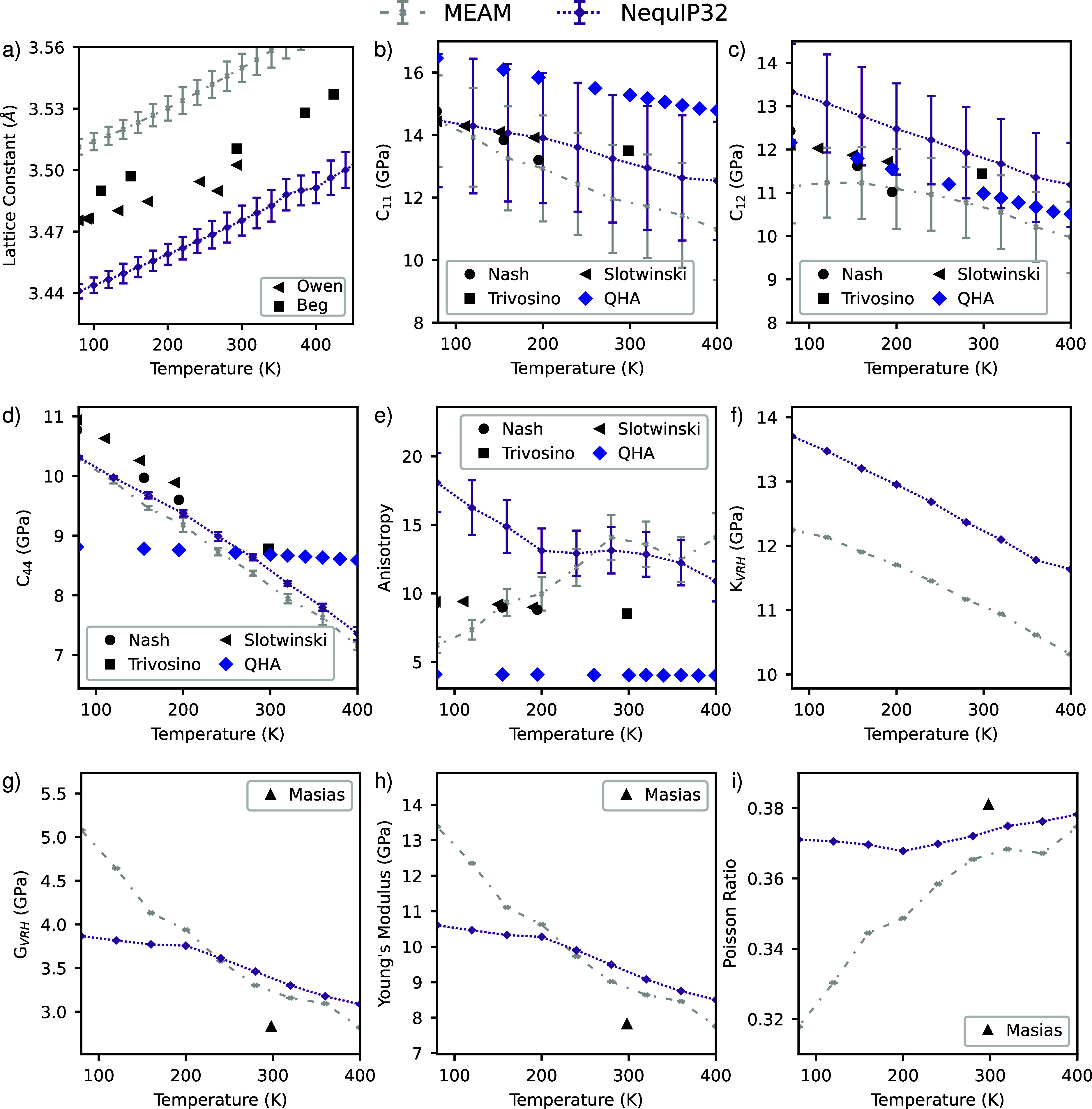
Bulk mechanical properties of lithium as a function of temperature calculated using the NequIP32 and MEAM potential and compared to the experimental results^38−41^ and the quasiharmonic approximation (QHA)^37^ where possible. (a) Lattice constant as a function of temperature with error bars as standard deviations of the volume fluctuation in the NPT simulation. (b–d) *C*_11_, *C*_12_, and *C*_44_ elastic constants, respectively, with error bars as the standard error from the fitting of stress–strain curves. Note how the QHA fails to capture the behavior of *C*_44_. (e) Elastic anisotropy with error bars propagated from errors in the elastic constants. (f–i) Voigt–Reuss–Hill averaged bulk, shear, and Young’s moduli and the Poisson ratio, respectively.

We computed a number of elastic properties using MD implemented in the LAMMPS with the potentials considered in this work. In Figure 2, we show the results of the NequIP32 and MEAM potential. Results for the SNAP and DP as well as the simulation protocols can be found in the Supporting Information.

The temperature dependence of the lattice constant of BCC lithium is shown in Figure 2a. The lattice constant is well captured within 1.5% by all of the potentials and is used to determine the volume used in the constant volume simulations for the elastic constants. The temperature range considered was chosen because there exists a Martensitic transition into an FCC structure at 78 K and the melting point of lithium is 450 K.^43^

The elastic response of single crystal BCC lithium, particularly at microscopic scales, is key to the design of LMBs. The bulk and shear moduli are parameters in the model of Monroe and Newman as well as the mode of Ahmad and Viswanathan used to predict stability against the formation of dendrites.^4,5^ Due to lithium’s low melting point, it is a soft material whose mechanical response can have unique properties near room temperature. The interplay between elastic and plastic regimes has been a topic of study.^37,42,44^ Xu et al. measured the elastic constants of lithium nanopillars and proceeded to calculate bulk elastic constants using a QHA within DFT.^37^ They found that the QHA performed poorly, hence the need for simulations at the fidelity of AIMD. A possible justification for the poor performance of the QHA is that it is only exact for harmonic potentials, which is only a good approximation near 0 K. The temperatures being explored in this study are on the order of ∼20–100% of the homologous temperature of lithium where harmonic approximations are less reliable for solids.

We perform the calculations for the elastic constants *C*_11_, *C*_12_, and *C*_44_ by fitting stress–strain curves following the prescription by Zhang et al.^45^ All other elastic constants for cubic crystals can be derived from these three using well-known formulas^46^ implemented in pymatgen.^35^ The predictions of *C*_11_, *C*_12_, and *C*_44_, the universal anisotropy, Voigt–Reuss–Hill (VRH) averaged bulk and shear moduli (*K*_VRH_ and *G*_VRH_, respectively), and the Young’s modulus and Poisson ratio are plotted as a function of temperature in Figure 2. The VRH average is a simple but reliable method for predicting polycrystalline elastic moduli given the relevant single crystal elastic constants.^46^

As shown in Figure 2, the NequIP32 MLIP reproduces the experimental results remarkably well. The NequIP32 outperforms the QHA and MEAM potentials in reproducing experimental results for single crystal lithium and in the prediction of VRH averaged quantities. The QHA is the only model that fails to predict *C*_44_ qualitatively accurately, underestimating the dependence of *C*_44_ as a function of temperature.

Overall, the NequIP32 is in excellent agreement with experimental results, consistently within 10% or less of the experimental results for *C*_11_, *C*_12_, and *C*_44_ and with matching qualitative behavior. The performance of the NequIP32 is attributed to the more accurate stress predictions. The only exception is the anisotropy, which is overestimated at low temperatures and shows a much larger decrease with increasing temperature than experimental predictions. A more thorough prediction of the anisotropy considering different crystal orientations could potentially improve the anisotropy prediction, but it is beyond the scope of this work. The NequIP32 potential in this work is the most accurate potential with which to calculate finite-temperature bulk and elastic phenomena for BCC lithium.

### 3.3 Adsorption Energies and Surface Diffusion Barriers

Surface properties such as adsorption and diffusion barriers are also key metrics in the LMB design. A number of studies predict these properties using DFT, but they are often forced to limit the surface area of the slabs, the Miller indices considered and the number of layers in their slab models.^6,47,48^ The workaround is to use slab models with 4–6 layers while fixing the bottom 2, artificially imposing a bulk environment close to the surface.^36^ With DFT, we found that it was impossible to converge the number of layers in the calculation of adsorption energy to within ∼1 meV/atom, even for the lowest Miller indices, with less than 10 layers, leading to reduced accuracy even with DFT. This makes the slab models necessary to calculate converged adsorption energies and surface diffusion barriers too large to calculate in DFT for higher Miller indices.

Since the MLIP is not limited as much as DFT in the number of layers and area, we can adequately converge calculated energies, typically on the order of tens of layers even with large surface areas. We can also go up to very high Miller indices, which are extremely costly or otherwise impossible to do with DFT.

**Figure 3:**
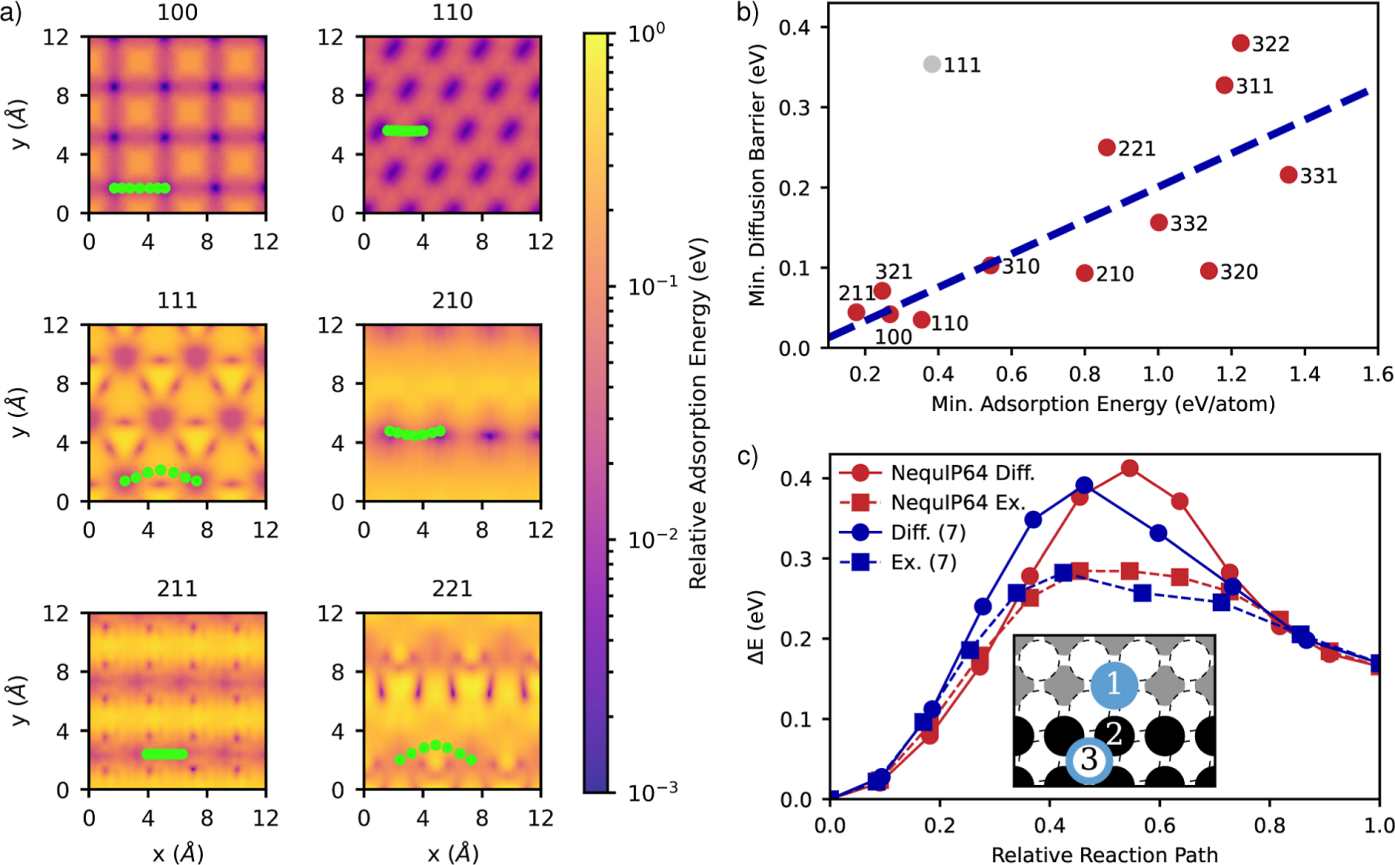
Various surface properties calculated using the NequIP64 potential. (a) Demonstration of a BEP correlation between the adsorption energy and the minimum diffusion barrier of each facet. The (111) surface was not included in the fitting of the dotted line. (b) Calculation of the Ehrlich–Schwöebel barrier matching results in the literature. (c) A variety of SPESs colored by the relative adsorption energy show the different geometries of adsorption sites and the minimum diffusion barrier paths adatoms can take when diffusing from one surface unit cell to the next.

For all results in this section, we use the most precise NequIP64 potential. We plot the surface potential energy surface (SPES) for various Miller indices in Figure 3a. The SPES is calculated by adsorbing a lithium atom on the relevant facet at various positions in the surface unit cell of a 4 × 4 surface supercell and allowing the adatom to relax only in the perpendicular direction (*z* direction) to the surface. All other atoms (except the 6 fixed layers at the bottom of the slab) are allowed to relax in any direction. The adsorption energy (*E*_ads_) is calculated using an unconventional bulk reference for the adatom as
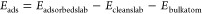


The bulk reference is chosen as it is more reliably predicted by all the potentials since no single atom data point was added to the training data sets, and we are only interested in relative adsorption energies and activation barriers. The SPES shows the diverse geometries and distributions of adsorption sites that can arise with exposure of different facets and can be used for thermodynamic lattice models of adsorption and surface diffusion. SPESs for higher Miller indices can be found in Figure S11.

We also calculate the surface diffusion barriers as an adatom diffuses from one surface unit cell to the next using the nudged elastic band (NEB) method with a spring constant of 0.1 eV/Å and 7 images as implemented in ASE.^49^ The paths with the lowest diffusion barriers identified by the NEB method are shown in the SPES in Figure 3a. We find that for all higher Miller indices considered, the lithium atoms are more likely to diffuse via one-dimensional channels at step edges as there are higher coordination numbers there in agreement with Gaissmaier et al. Only (100), (110), and (111) have equally high diffusion barriers in two dimensions.^7^

In Figure 3b, we plot the adsorption energy versus the lowest surface diffusion barrier for that facet and show that there exists a linear correlation between the adsorption energy and the surface diffusion barrier. This is an example of a Bell–Evans–Polanyi (BEP) principle, a general framework that notes that the difference in activation energy between two reactions of the same family is roughly proportional to the difference of their enthalpy of the reaction.^50,51^ In the context of adsorption energies and surface diffusion barriers, Pande and Viswanathan found a BEP relation relating the adsorption energies on low Miller index facets for different metals and alloys and the surface diffusion barriers on those surfaces.^48^ The BEP relation can be used as a powerful screening tool that avoids expensive NEB calculations. We show that there also exists a BEP relation for different Miller indices of the same material, which can be used for screening purposes in cases where the NEB method is too costly. We note that the (111) surface is an outlier to the BEP relation due to its very high surface energy as it is not a close-packed surface for the BCC crystal structure and all the surface atoms are under-coordinated.

We also predict an example of the Ehrlich–Schwöebel barrier, a descriptor used in determining the likelihood of an adatom to diffuse up a step edge.^52^ The activation energy for this process is increased as the adatom goes from a region of high coordination along the step edge to a region of lower coordination on the top of the step. The Ehrlich–Schwöebel activation barrier (*E*_S_) is defined as *E*_S_ = *E*_ESB_ – *E*_T_, where *E*_ESB_ is the increased barrier due to having to diffuse up a step edge and *E*_T_ is the activation energy for diffusion without any steps.^7^ We consider two mechanisms for step-up diffusion on the most stable (100) surface and show in Figure 3c that NequIP64 reproduces the results by Gaissmaier et al. to within 0.01 eV for the activation energy for both mechanisms.^7^ A schematic to explain the two mechanisms is shown in Figure 3c. The first mechanism is when the adatom directly diffuses (Diff.) over the step edge (1 → 3) and the second is by exchanging (Ex.) with an atom in the step (1 → 2, 2 → 3).

Overall, the NequIP64 potential has shown very good accuracy in reproducing and predicting surface properties and is available to the community. For novel applications beyond the scope of this work, it still remains important to benchmark the MLIPs. This is particularly true in cases where the simulations being performed are unphysical or sample out-of-domain data. For specific simulations, we advise performing DFT calculations on a few structures sampled from the MLIP simulation using the provided DFT parameters in the domain of interest for comparison. A major advantage of MLIPs over empirical potentials is that if it is found that the MLIP is out of domain and has a high error, new data in the relevant domain can be added to easily retrain the students and thereby improve the potential. The current provided data set provides a base for continually improving potentials. In this work, the MLIPs have allowed the prediction of potential energy surfaces and diffusion barriers that are computationally infeasible using DFT and established the existence of a BEP relation across different facets.

## 4 Discussion: Implications for LMB Design

The calculations enabled by the lithium MLIPs derived in this work now allow the refining criterion for morphological stability associated with dendrite formation described above. Monroe and Newman and, subsequently, Ahmad and Viswanathan showed that room-temperature shear modulus and anisotropy of lithium are critical factors in determining the stability criterion for dendrite suppression and thereby the required shear modulus of the solid electrolyte.^4,5^ In this work, we show that the temperature-dependent response of the mechanical properties is much stronger than that previously predicted using the QHA. The room-temperature modulus, as predicted by NequIP32 is around 3.5 GPa, a reduction of about ∼30% from that at 0 K using the QHA, drastically modifying the stability criterion, thereby modifying the required shear modulus of the solid electrolyte needed to suppress dendrite formation by a similar ∼30%.

Jäckle et al. and Gaissmaier et al. probed surface diffusion barriers for low Miller index facets, tractable using DFT calculations.^6,7^ Rapidly evolving morphologies can locally have high Miller index domains with a high degree of undercoordination. Using the lithium MLIP developed here, we show the existence of a BEP relation, indicating that high Miller indices, which typically have higher lithium adsorption energy due to undercoordination, have much larger surface diffusion barriers. For the (110) and (211) facets that appear in the Wulff construction, the diffusion rates ν ∝ exp(−Δ*E*/*k*_B_*T*) at 300 K are 1–2 times slower than those on the (100) facet with the lowest barrier. The exception is the (111) surface, which has an anomalously large surface diffusion barrier with respect to the BEP relation. Other facets that do not appear in the Wulff construction all have higher diffusion barriers with ν > 4 times that of the (100) surface or many orders of magnitude more, even along preferential one-dimensional channels observed in the SPES.

Another implication is that glassy phases that have been shown to improve bulk conductivity^32^ are likely to possess lower surface diffusion, limiting the charging rate when glassy phases of lithium are formed. Surface diffusion in glassy phases is further hampered by the lack of long-range order as lithium is deposited, which would randomly orient the preferential one-dimensional channels with high adsorption energy, thereby trapping atoms in DP wells. We therefore expect that the combined effect is the proliferation of local instabilities under fast charging conditions before the surface equilibrates to (100) or (110) facets with faster diffusion in two dimensions, which would reduce dendrite growth.

We believe that the lithium MLIP and training data set developed here will enable the calculation of void formation, creep behavior, and various other mesoscale properties previously not tractable using atomistic simulations with significantly improved accuracy, thereby allowing more refined material properties under different conditions.

## Data Availability

The potentials and supporting data set used in this work are made available on Zenodo (2024). doi: 10.5281/zenodo.10470793.

## Supporting Information Available

Sata set, model parameters and simulation methods, data set distribution, additional benchmarks, elastic constant data for other potentials, and more facets (PDF)
