# Supplementary material for: Accurate Surface and Finite-Temperature Bulk Properties of Lithium Metal at Large Scales Using Machine Learning Interaction Potentials
Source: ACS Omega. 2024 Feb 21;9(9):10904–12. doi: 10.1021/acsomega.3c10014 (PMC10918842; doi:10.1021/acsomega.3c10014)
Supplement: Supplementary file 1 — ao3c10014_si_001.pdf [file ao3c10014_si_001.pdf]

Supporting Information For: Accurate Surface and Finite Temperature Bulk Properties of Lithium Metal at Large Scales using Machine Learning Interaction Potentials

Mgcini Keith Phuthi,[†] Archie Mingze Yao,[†] Simon Batzner,[‡] Albert Musaelian,[‡] Pinwen Guan,[†] Boris Kozinsky,[‡] Ekin Dogus Cubuk,[¶] and Venkatasubramanian Viswanathan[∗,†,§]

†*Department of Mechanical Engineering, Carnegie Mellon University, Pittsburgh, PA, USA*

‡*School of Engineering and Applied Science, Harvard University, Cambridge, MA, USA*

¶*Google Research, Brain Team*

§*Corresponding Author*

E-mail: venkvis@cmu.edu

MLIP hyperparameters

The full training and test datasets and configuration files used to train the NequIP and DP models can be found in the zenodo data storage. Here we comment on some key hyperparameter choices made in the NequIP model.

Dataset composition

A break-down of the seed structures used to generate the dataset and the number of structures that ended up in the dataset are given in Table 1 and the distribution in the dataset is given in Fig. S1, Fig. S2 and Fig. S3

Table S1. Distribution of seed structures used in dataset. It is worth noting that the MD simulations used to generate the data were run at varying pressure, most data was collected from molten structures above the melting point, allowing robust exploration of phase space. Extrapolation tests on other crystal structures, surfaces and grain boundaries showed that it was not necessary to add more data for these structures.

Structure type	Run name	Seed structures	Number of datapoints	Num. of atoms
Bulk	run1	bcc, fcc, hcp	306	128, 108
	run2	bcc, fcc, hcp	591	2,4
	run3	bcc, hcp	227	16,32
	run4	fcc	116	54
	run5	fcc	279	54
	run6	bcc, fcc, hcp	186	128
	Total		1705	
Vacancies	runv0	bcc, fcc, hcp	18	107, 127
	runv1	bcc, fcc, hcp	77	107, 127
	Total		187	
Interstitials	runi0	bcc, fcc, hcp	92	109, 129
(All sites)	runi1	bcc	196	129
	runi2	fcc	182	109
	runi3	bcc, fcc, hcp	55	129
	Total		433	
Slabs	runsurf1	bcc 100, 110, 111	27	12
	runsurf2	bcc 100, 110, 111	40	128
	runsurf3	bcc 100, 110, 111 (adsorbed)	405	81, 129, 133, 121
	runsurf4	bcc 100, 110, 111	664	162, 167, 135, 118, 122
	runsurf5	bcc 100, 110, 111	593	160, 114, 116, 122, 124
	runsurf6	bcc 210, 211, 221	328	145, 161
	runsurf7	bcc 210, 211, 221	450	145, 161
	runsurf8	bcc 111	221	161, 162, 163, 168, 169, 158
	Total		2728	
	Grand total		5053	

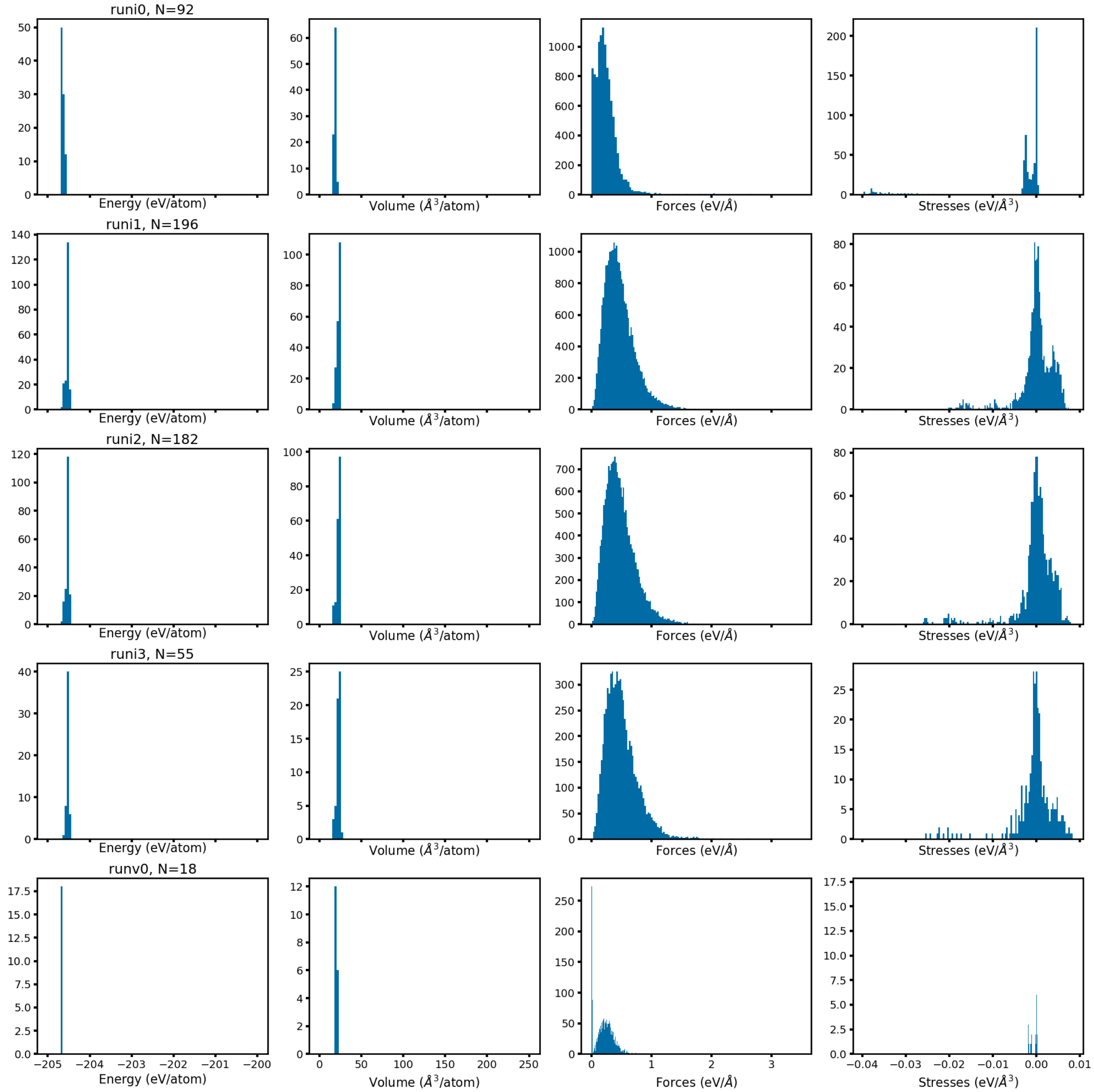

Fig. S1. Distribution of energies, volumes, forces and stresses in the dataset.

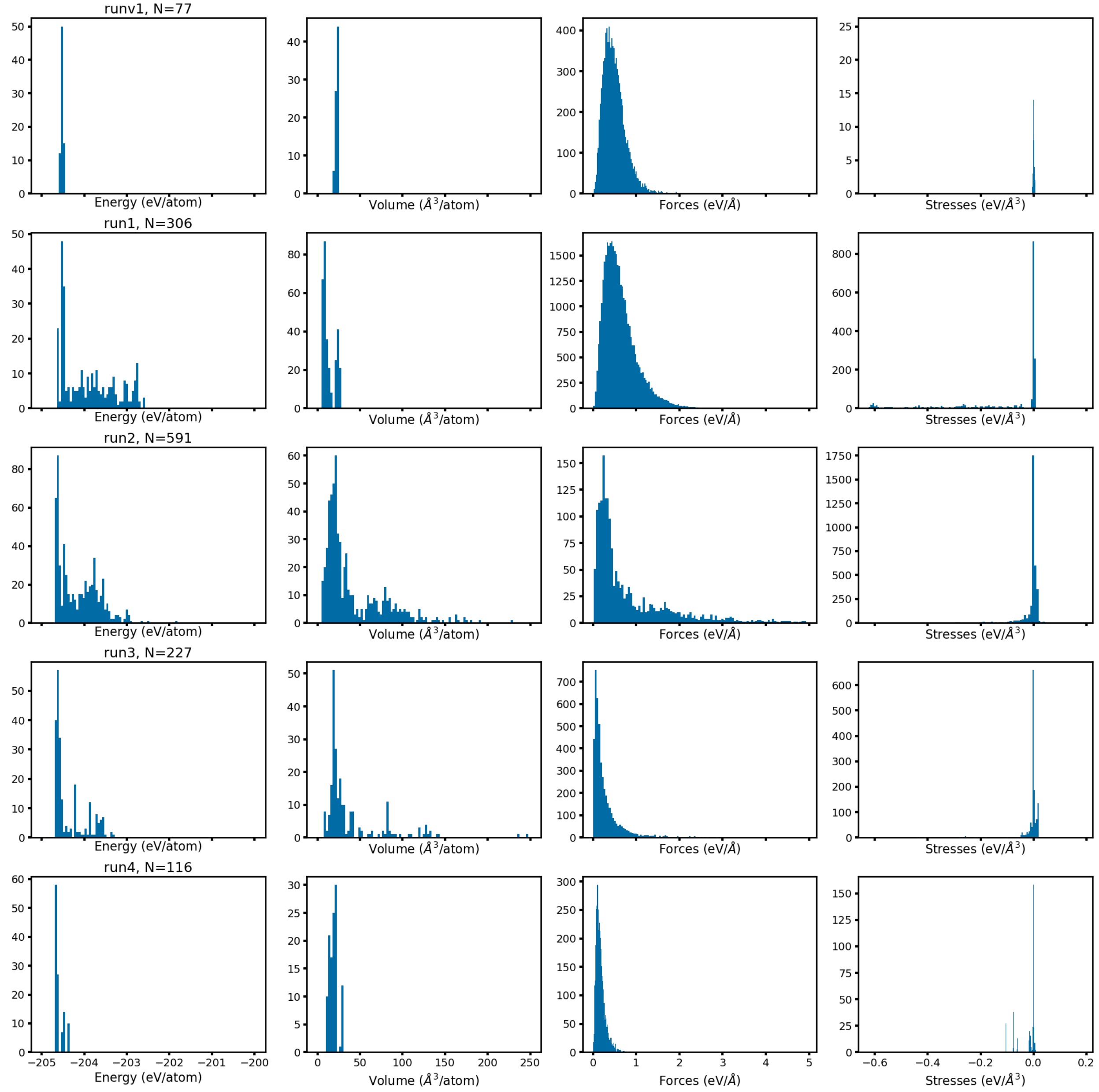

Fig. S2. Distribution of energies, volumes, forces and stresses in the dataset.

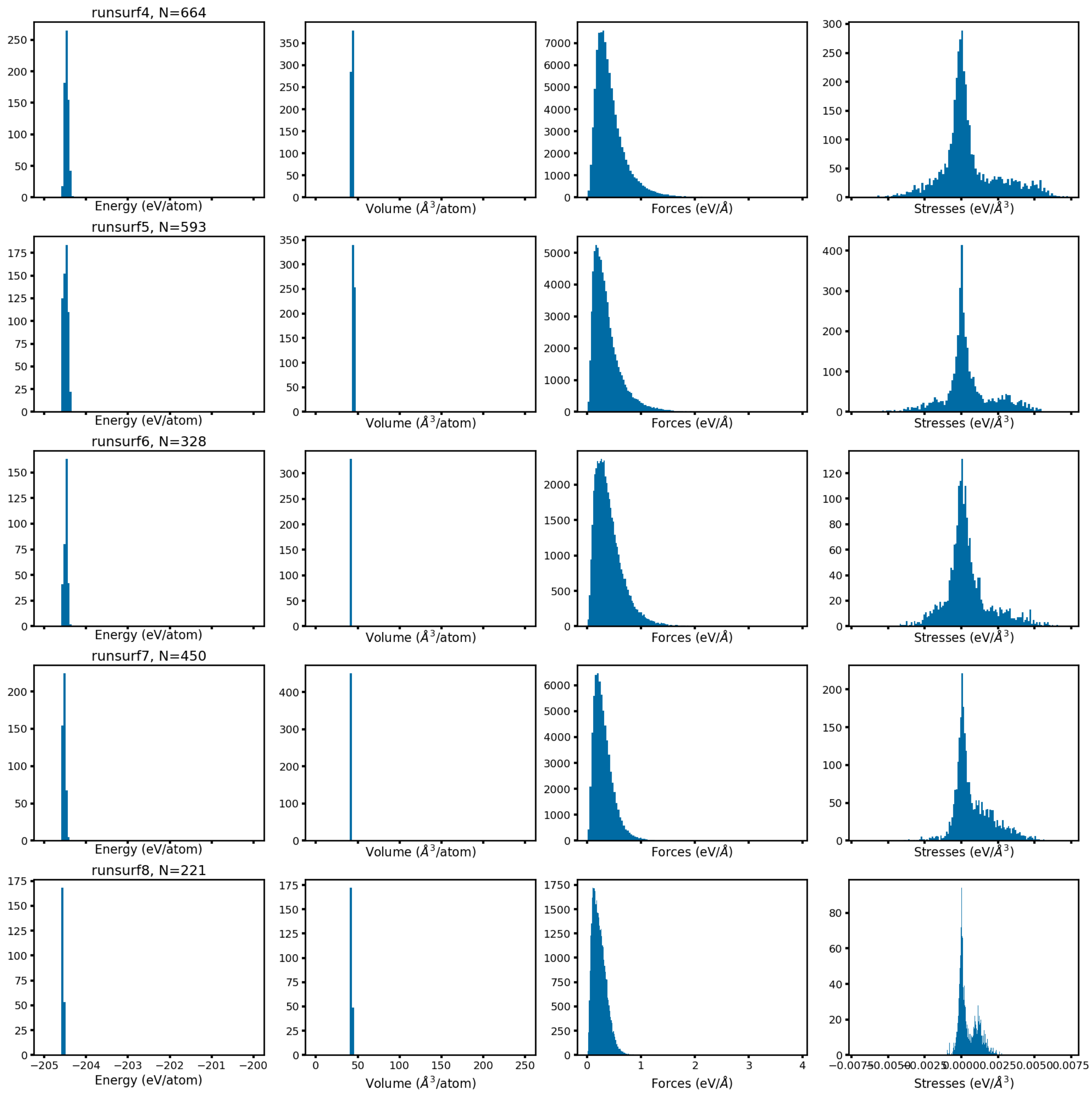

Fig. S3. Distribution of energies, volumes, forces and stresses in the dataset.

Loss function

The loss function used in the model is a typical sum of Mean Square Errors of energy, force and stress loss given by

$$\mathcal{L} = \beta_e \frac{1}{N} \sum_{i}^{N} ||\hat{E}_i - E_i||^2 + \beta_f \frac{1}{3N} \sum_{i}^{N} \sum_{\alpha=1}^{3} ||\hat{f}_{i,\alpha} - f_{i,\alpha}||^2 + \beta_\sigma \frac{1}{6N} \sum_{i}^{N} \sum_{j}^{6} ||\hat{\sigma}_{i,j} - \sigma_{i,j}||^2. \quad (1)$$

The ground-truth energy, force components and stress components are E_i, $f_{i,\alpha}$ and $\sigma_{i,j}$ for the i-th atom. The hatted symbols are the MLIP predictions. The key hyperparameters in the loss function are the loss coefficients $\beta_e = 10$, $\beta_f = 1$ and $\beta_\sigma = 1000$ for weighting the energy, force and stress loss respectively found after hyperparameter tuning. The larger focus on stress is due to the smaller values that stress typically takes, increasing β_σ significantly improved stress accuracy without negatively affecting energy and force accuracy.

Floating point precision

The choice of whether to train and deploy the potentials with single (float32) or double (float64) floating point precision affects the memory footprint of the models and precision. Ideally, the lowest precision would be used but lower precision comes at the cost of loss in accuracy for particular calculations. Calculations involving small differences in extensive quantities such as energy between systems differing in numbers of atoms are particularly prone to numerical errors as they may have different mantissas larger than the precision required. As an example, we show the calculation of the vacancy formation energy of BCC lithium with different potentials. The vacancy formation should converge with increasing system size in Fig. S8 but due to the precision required being smaller than the mantissa, the NequIP32 model predicts fluctuating values. This has been resolved by changing the last layers of the neural network to be in double precision even when the model parameters are in single precision in future models of NequIP.

For calculations dependant on force and stress however, we did not see any significant numerical differences as they are intensive. Hence we use the NequIP32 potential implemented in LAMMPS to perform MD simulations faster. For the surface calculations, we use the the NequIP64 model.

SNAP

The details of the SNAP potential are described by Zuo et al.[1] We also note that we trained the NequIP architecture on the data that was used to train the SNAP potential and got better test

errors on the SNAP test set with little hyperparameter tuning. NequIP RMSEs were 1.3meV/atom and 15mev/Å vs quoted SNAP RMSEs of 1.4meV/atom and and 40meV/Å for SNAP for energy and force RMSE respectively.[1] We thereby expect that the NequIP architecture is generally more accurate for the same dataset, consistent with previous results.[2]

Deep Potential

Deep Potential provides a trainable, fully local framework for training MLIPs that can predict energy, forces and stresses of a particular configuration of atoms. In this work, we choose the se_e3 descriptor constructed from radial and angular information 18 of atomic environments. This descriptor is invariant to the symmetries and not trainable. The neural network architecture is that of Residual Neural Network.

48 MLIP benchmarks

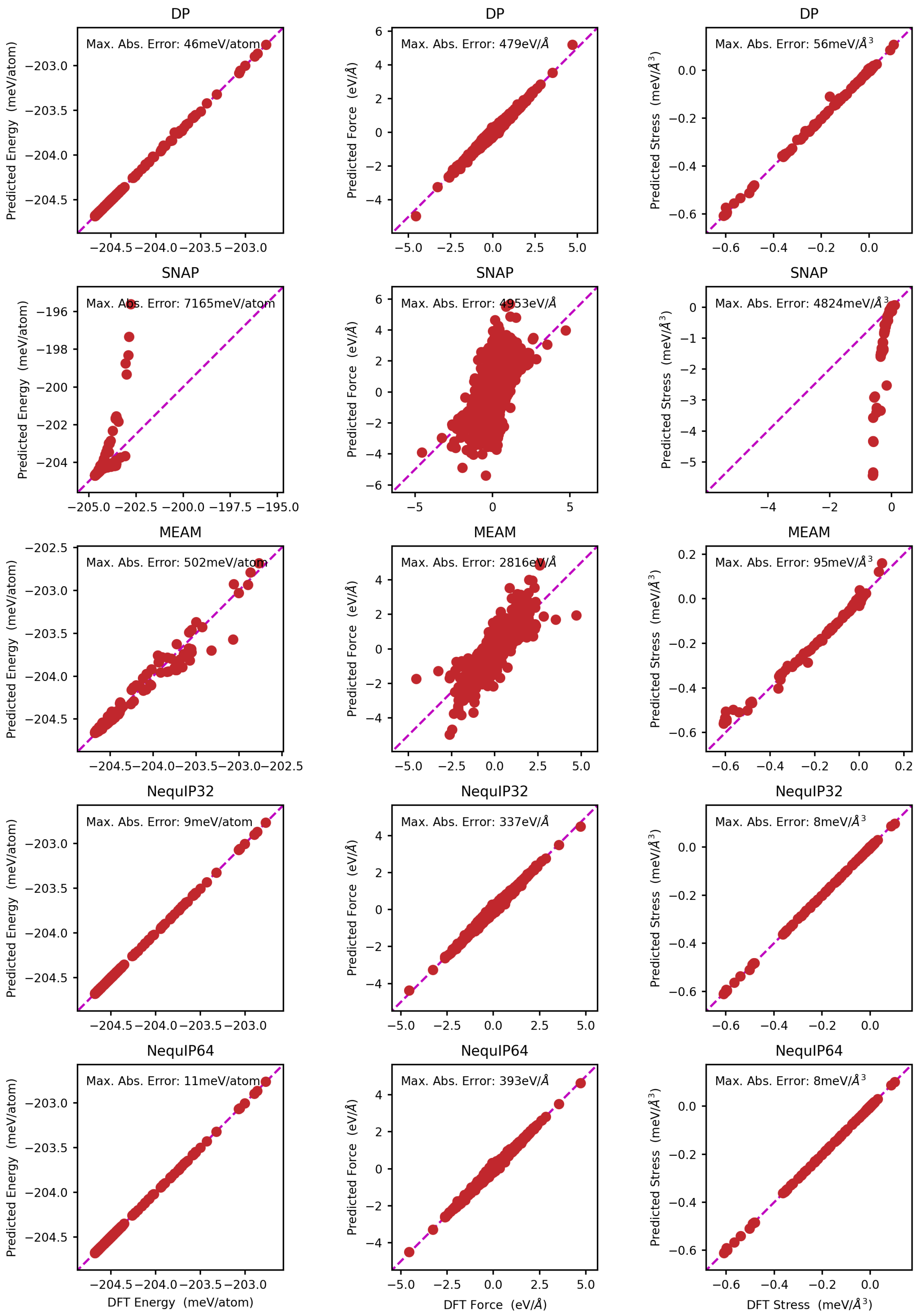

Fig. S4. Parity plots for the various potentials relative to DF predicitons on the test set.

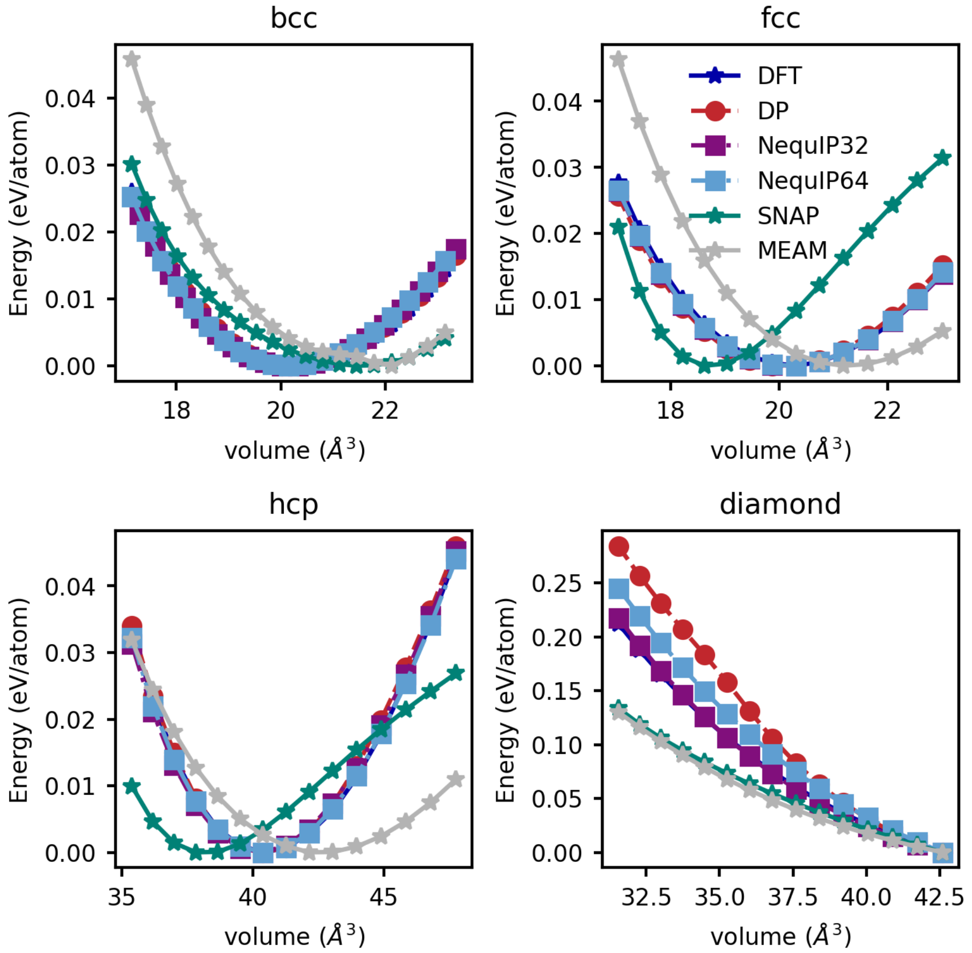

Fig. S5. Energy vs Volume plots for FCC, BCC, HCP and Diamond structures

Cost vs Accuracy

To give a sense of the relative cost versus accuracy of the different models, we plot the force and stress RMSE relative to the DFT test dataset in Fig. S6 and Fig. S7 respectively. The cost is evaluated as the time taken to evaluate the energy, force and stress of a 128 atom unit cell with the converged parameters. This is only a rough estimate as the computation cost scales differently with the number of atoms with each method.

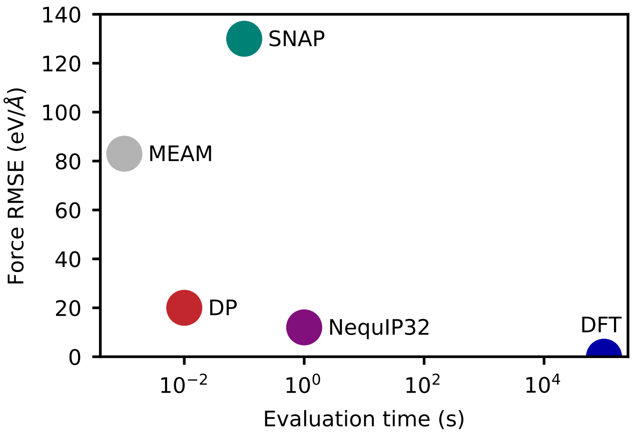

Fig. S6. Force error vs computational speed for one evaluation of a unit cell with 128 atoms.

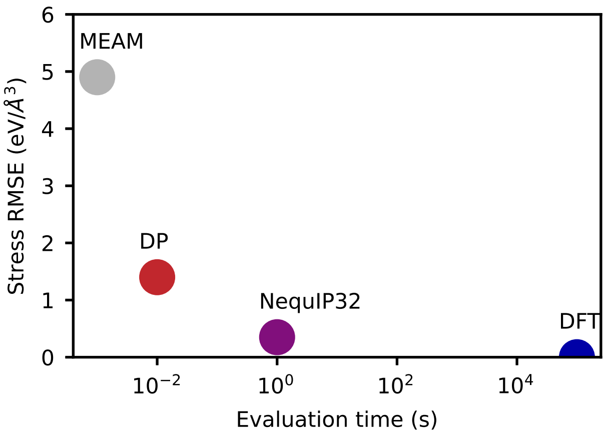

Fig. S7. Stress error vs computational speed for one evaluation of a unit cell with 128 atoms. Notice how NequIP significantly outperforms the other models in accuracy. The SNAP error of 259.7meV/Å^3 is not included for clarity.

Methodology of Calculations

Monovacancy Formation Energy(E_v) at 0K

E_v is calculated using the formula

$$E_v = E_{\text{bulk with vacancy}} - \frac{N-1}{N} E_{\text{bulk without vacancy}}$$

where N is the number of atoms in the bulk structure without any vacancies. The reported value is that for a cubic BCC supercell of size 5x5x5, the maximum size we calculated with DFT. The convergence of the vacancy formation energy with increasing supercell size is shown in Fig. S8

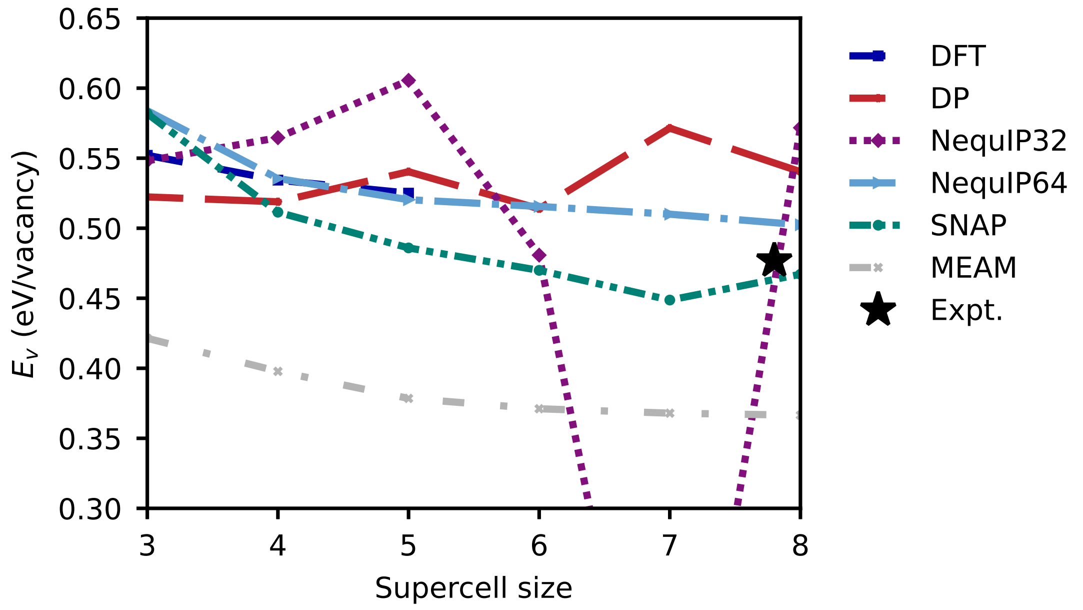

Fig. S8. Vacancy formation as a function of supercell size. The experimental value is from[3]

Elastic Constants at 0K

The elastic constants were calculated using the same method as Ahmad et al. (29) as an expansion of the energy about the equilibrium position with different volume conserving strains. The free energy F can be expanded as a function of the strains e_i in Voigt notation as

$$F = F_0 + \frac{V}{2} \sum_{i=1}^{6} \sum_{j=1}^{6} C_{ij} e_i e_j + O(e_i^3) \tag{2}$$

where F_0 is an arbitrary constant and $C_i j$ are the components of the elastic tensor. In practice since we are only predicting the 0K elastic constants, the DFT energy is equivalent to the Free Energy. Combinations of the elastic constants can therefore be extracted as in any standard textbook.

In our work we use the bulk modulus

$$K = (C_{11} + 2C_{12})/3 \tag{3}$$

which is obtained by fitting the Birch-Murnaghan equation of state. Additionally, the volume-conserving orthorhombic strain with $e_1 = -e_2 = x$, $e_3 = x^2/(4 - x^2)$ and $e_4 = e_5 = e_6 = 0$ to get

$$C' = C_{11} - C_{12}. \tag{4}$$

Eq. 3 and eq. 4 can be solved to isolate C_{11} and C_{12}. Finally, the volume-conserving monoclinic strain with $e_4 = x$, $e_1 = x^2/(4 - x^2)$ and $e_2 = e_3 = e_5 = e_6 = 0$ is used to find C_{44}.

Finite Temperature Elastic Constants

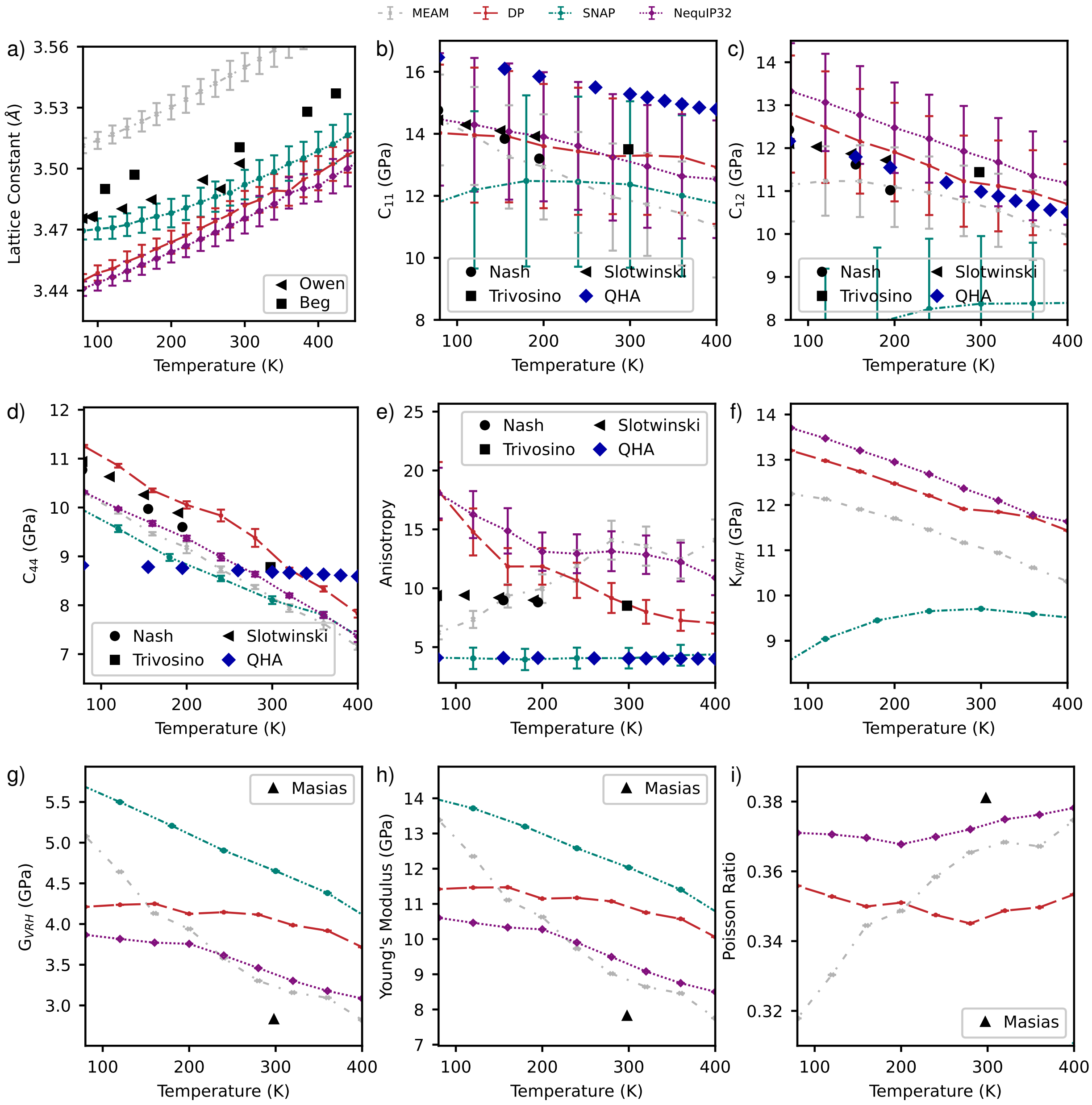

Fig. S9. Bulk mechanical properties of lithium as a function of temperature calculated using NequIP32, DP, MEAM and SNAP potentials and compared to experiment and the Quasiharmonic Approximation (QHA)[4] where possible. a) Lattice constant as a function of temperature with error bars as standard deviations of the volume fluctuation in the NPT simulation. (b-d) C_{11}, C_{12} and C_{44} elastic constants respectively with error bars as standard error from the fitting of stress-strain curves. Note how the QHA fails to capture the behavior of C_{44}. (e) The elastic anisotropy with error bars propagated from errors in the elastic constants. (f-i) Voigt-Reuss-Hill averaged Bulk, shear, Young's modulus and the Poisson ratio respectively.

The temperature dependence of the lattice constant of BCC lithium is shown in Fig. S9a.

Starting from the conventional equilibrium BCC structure in a 6x6x6 supercell at 0K for each potential, an NPT simulation is used to raise the temperature at a heating rate of 0.01K/timestep and then run at equilibrium for 100,000 timesteps with 1fs/timestep at temperature T and zero external stress allowing the volume of the simulation box to change while keeping the unit cell orthorhombic. The average box size for the last 80,000 timesteps sampled every 100 timesteps can be used to extract the average lattice constant as a function of temperature.

All the potentials perform well with less than 1.5% error for the lattice constant with a slight underestimation for the MLIPs. The predictions using other potentials are shown in the Supporting Information (SI Appendix). The temperature range considered was chosen because there exists a Martensitic transition into a FCC structure at 78K and the melting point of lithium is 450K.[10]

The elastic response of single crystal BCC lithium, particularly at microscopic scales is key to the design of LMSSBs. The bulk and shear modulus are parameters in the model of Monroe and Newman as well as Ahmad and Viswanathan used to predict stability against the formation of dendrites.[11,12] Due to lithium's low melting point, it is a soft material whose mechanical response can have unique properties near room temperature. The interplay between elastic and plastic regimes has been a topic of study.[4,9,13] Here, we calculate the elastic constants of single crystal BCC lithium as a function of temperature. Xu et al. measured the elastic constants of lithium nanopillars and proceeded to calculate bulk elastic constants using a Quasiharmonic approximation (QHA) within DFT.[4] They found that the QHA performed poorly hence the need for simulations at the fidelity of AIMD.

We perform the calculations for the elastic constants C_{11}, C_{12} and C_{44} by fitting stress-strain curves after applying two different types of strain, an orthorhombic and monoclinic strain and perform 1ns long NVT simulations following the prescription by Zhang et al.[14] to save computational cost. All other elastic constants for cubic crystals can be derived from these three using well known formulae[15] implemented in pymatgen.[16]

The predictions of the components of the C_{11}, C_{12} and C_{44}, the Universal Anisotropy, Voigt-Reuss-Hill averaged bulk and shear moduli (K_{VRH} and G_{VRH} respectively) as well as the Young's

modulus and Poisson Ratio are plotted as a function of temperature in Fig. S9. The Voigt-Reuss-Hill (VRH) average is a reliable method for predicting polycrystalline elastic moduli given the relevant single crystal elastic constants while being very easy to compute.[15]

As shown in Fig. S9, the MLIPs in this work reproduce the experimental results remarkably well, significantly outperforming the QHA, SNAP and MEAM in reproducing experimental results for single crystal lithium and in the prediction of VRH averaged quantities.

The QHA is the only model that fails to predict the C_{44} qualitatively accurately underestimating the dependence of C_{44} as a function of temperature, potentially due to the assumed independence of vibrational modes in each spatial dimension.

NequIP32 is in excellent agreement with experimental results, consistently within 10% or less of the experimental results for C_{11}, C_{12} and C_{44} and with matching qualitative behavior. The performance of NequIP32 is attributed to the more accurate stress predictions. The only exception is the Anisotropy which is overestimated by DP and NequIP32 at low temperatures and shows much larger decrease with increasing temperature than experimental predictions. Overall, the NequIP32 potential in this work is the most accurate potential with which to calculate bulk and elastic phenomena for BCC lithium.

Surface Energies and Wulff Shape

As shown in Fig. S10a, in addition to bulk properties, the NequIP potentials accurately describe the surface energies of lithium. Surface and adsorption energies are often quoted up to $\sim$10meV/Å^2 precision due to finite size effects and propagation of errors. The NequIP potentials are well within 1meV/Å^2 error for all the miller indices despite being only explicitly trained on the (100), (110) and (111) planes as starting seeds. This demonstrates the excellent generalization to higher miller index surfaces which we take advantage of in the calculation of surface properties in Fig. S11 and Figure 2. The predicted surface energies for low miller indices listed in Table 1 agree very well with both our DFT and DFT results in other works as well.

We also calculate the 0K Wulff construction which determines the equilibrium shape of a

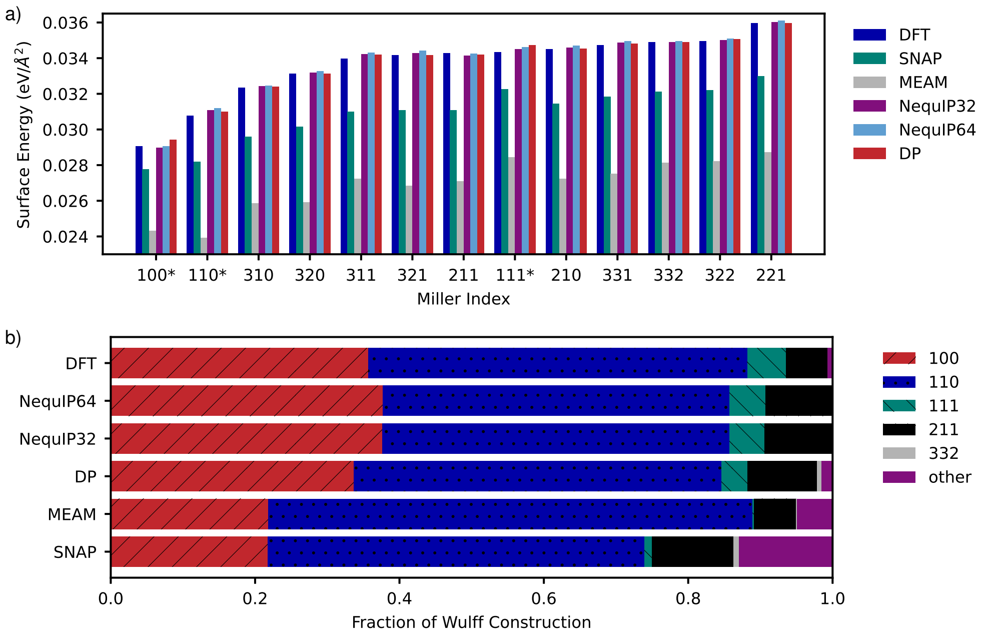

Fig. S10. Various surface properties of BCC lithium calculated and compared with DFT results. a) Surface energies for various facets of BCC lithium. NequIP and DP potentials reproduce DFT results very well while MEAM and SNAP have large errors relative to the DFT prediction. b) The Wulff construction for BCC lithium showing that BCC lithium in vacuum is dominated by the (100), (110), (111) and (211) facets according to DFT. NequIP and DP potentials in this work reproduce that result but SNAP and MEAM predict a significant contribution from other facets and much less from (111).

droplet or crystal suspended in a medium which in this case is vacuum.[17] Depending on the surface energies and geometry, the shape of the droplet determines what fraction of the total area of the droplet is contributed by each facet. This allows estimation of the importance of particular facets in simulations at equilibrium. In the presence of an electrolyte, the Wulff construction might change due to the sensitivity to the small energy differences between higher miller indices and in practice the surrounding medium.[18] We follow the procedure used by Tran et al.[18] implemented in the Python Materials Genomics package (pymatgen) to estimate the area fractions for miller indices up to a value of 3 in Fig. S10b. The NequIP results agree well with the DFT prediction that (110) and (100) planes followed by (111) and (211) to a lesser extent dominate the Wulff construction. DP has slightly different results with (211) more dominant over (111) while MEAM and SNAP have much more significant differences as expected due to the poor prediciton of surface energies. The domination of (110) in the Wulff construction despite (100) having the lowest surface energy

is consistent with experimental results.[19]

The excellent reproduction of DFT calculated properties gives confidence that the MLIPs, particularly NequIP are truly reproducing the DFT result for surface and bulk properties with small errors at the level of typical DFT precision. We therefore assume subsequent errors are from the quality of the dataset, most likely the choice of exchange-correlation functional when comparing with experiment. In the rest of the paper, we demonstrate the superior accuracy in reproducing experimental results of the NequIP potentials over a range of properties that are difficult to predict using DFT.

Surface Potential Energy Surfaces

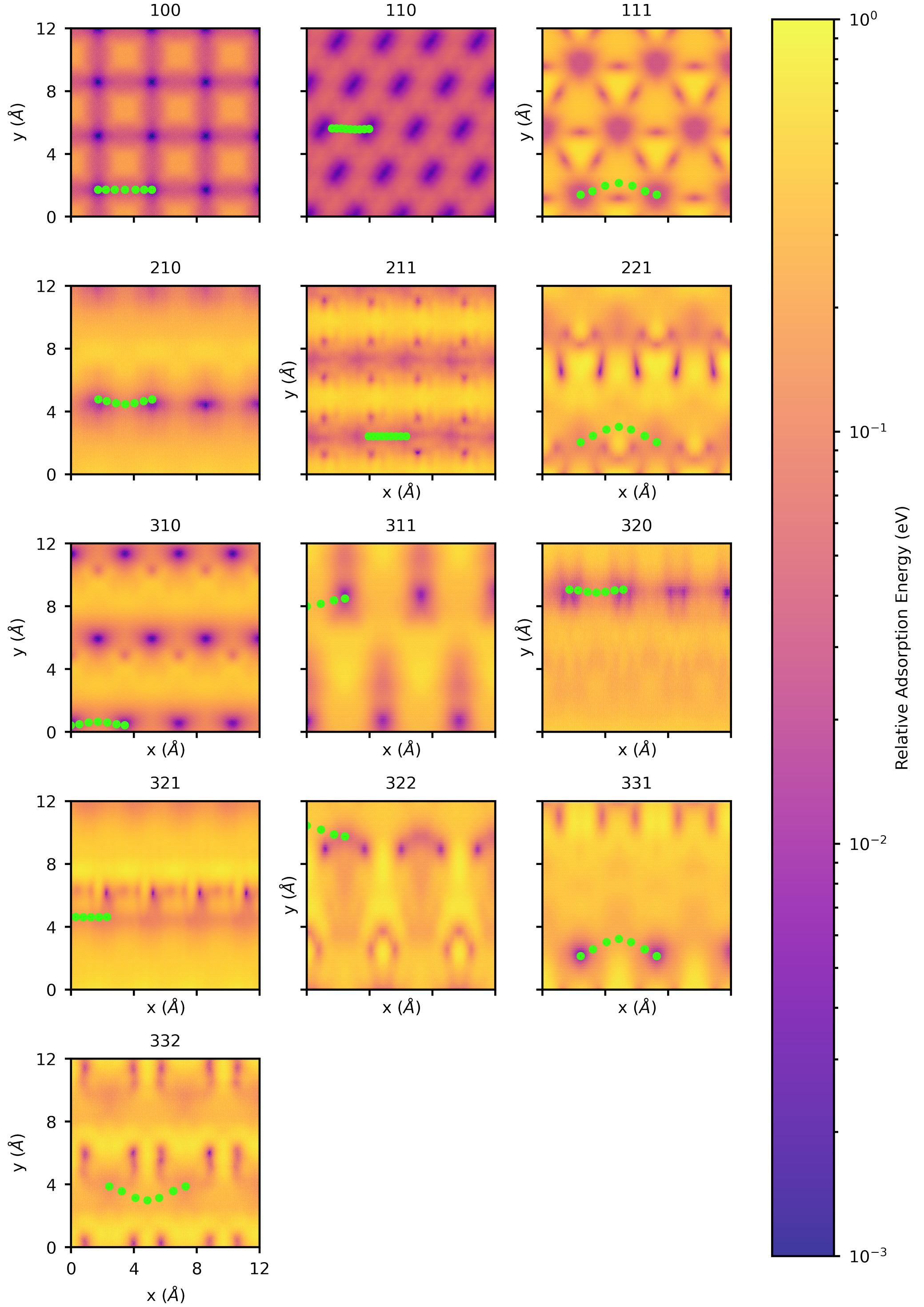

Fig. S11. Surface Potential Energy Surfaces for Miller indices up to 3 for BCC lithium

References

(1) Zuo, Y.; Chen, C.; Li, X.; Deng, Z.; Chen, Y.; Behler, J.; Csányi, G.; Shapeev, A. V.; Thompson, A. P.; Wood, M. A.; Ong, S. P. Performance and Cost Assessment of Machine Learning Interatomic Potentials. *The Journal of Physical Chemistry A* **2020**, *124*, 731–745, Publisher: American Chemical Society.

(2) Batzner, S.; Musaelian, A.; Sun, L.; Geiger, M.; Mailoa, J. P.; Kornbluth, M.; Molinari, N.; Smidt, T. E.; Kozinsky, B. E(3)-equivariant graph neural networks for data-efficient and accurate interatomic potentials. *Nature Communications* **2022**, *13*, 2453, Number: 1 Publisher: Nature Publishing Group.

(3) Starodubtsev, Y. N.; Tsepelev, V. S.; Wu, K. M.; Kochetkova, Y. A.; Tsepeleva, N. P. Vacancy Formation Energy of Metals. *Key Engineering Materials* **2020**, *861*, 46–51, Conference Name: Advanced Materials and Engineering Materials IX ISBN: 9783035716696 Publisher: Trans Tech Publications Ltd.

(4) Xu, C.; Ahmad, Z.; Aryanfar, A.; Viswanathan, V.; Greer, J. R. Enhanced strength and temperature dependence of mechanical properties of Li at small scales and its implications for Li metal anodes. *Proceedings of the National Academy of Sciences* **2017**, *114*, 57–61, Publisher: National Academy of Sciences Section: Physical Sciences.

(5) Owen, E. A.; Williams, G. I. X-Ray Measurements on Lithium at Low Temperatures. *Proceedings of the Physical Society. Section A* **1954**, *67*, 895–900.

(6) C. Nash, H.; Smith, C. S. Single-crystal elastic constants of lithium. *Journal of Physics and Chemistry of Solids* **1959**, *9*, 113–118.

(7) Trivisonno, J.; Smith, C. S. Elastic constants of lithium-magnesium alloys. *Acta Metall.* **1961**, *9*, 1064–1071.

(8) Slotwinski, T.; Trivisonno, J. Temperature dependence of the elastic constants of single crystal lithium. *Journal of Physics and Chemistry of Solids* **1969**, *30*, 1276–1278.

(9) Masias, A.; Felten, N.; Garcia-Mendez, R.; Wolfenstine, J.; Sakamoto, J. Elastic, plastic, and creep mechanical properties of lithium metal. *Journal of Materials Science* **2019**, *54*, 2585–2600.

(10) Beg, M. M.; Nielsen, M. Temperature Dependence of Lattice Dynamics of Lithium 7. *Physical Review B (Condensed Matter and Materials Physics)* **1976**, *14*, 4266–4273.

(11) Monroe, C.; Newman, J. The Impact of Elastic Deformation on Deposition Kinetics at Lithium/Polymer Interfaces. *Journal of The Electrochemical Society* **2005**, *152*, A396, Publisher: IOP Publishing.

(12) Ahmad, Z.; Viswanathan, V. Stability of Electrodeposition at Solid-Solid Interfaces and Implications for Metal Anodes. *Physical Review Letters* **2017**, *119*, 056003, Publisher: American Physical Society.

(13) Wang, Y.; Dang, D.; Wang, M.; Xiao, X.; Cheng, Y.-T. Mechanical behavior of electroplated mossy lithium at room temperature studied by flat punch indentation. *Applied Physics Letters* **2019**, *115*, 043903, Publisher: American Institute of Physics.

(14) Zhang, H.; Li, C.; Djemia, P.; Yang, R.; Hu, Q. Prediction on temperature dependent elastic constants of "soft" metal Al by AIMD and QHA. *Journal of Materials Science & Technology* **2020**, *45*, 92–97.

(15) Nye, J. F. *Physical Properties of Crystals: Their Representation by Tensors and Matrices*; Clarendon Press, 1985.

(16) Jain, A.; Ong, S. P.; Hautier, G.; Chen, W.; Richards, W. D.; Dacek, S.; Cholia, S.; Gunter, D.; Skinner, D.; Ceder, G.; Persson, K. A. Commentary: The Materials Project: A materials

genome approach to accelerating materials innovation. *APL Materials* **2013**, *1*, 011002, Publisher: American Institute of Physics.

(17) Balluffi, R. W.; Allen, S. M.; Carter, W. C. *Kinetics of Materials*, 1st ed.; Wiley-Interscience: Hoboken, N.J, 2005.

(18) Tran, R.; Xu, Z.; Radhakrishnan, B.; Winston, D.; Sun, W.; Persson, K. A.; Ong, S. P. Surface energies of elemental crystals. *Scientific Data* **2016**, *3*, 160080, Number: 1 Publisher: Nature Publishing Group.

(19) Li, Y.; Li, Y.; Pei, A.; Yan, K.; Sun, Y.; Wu, C.-L.; Joubert, L.-M.; Chin, R.; Koh, A. L.; Yu, Y.; Perrino, J.; Butz, B.; Chu, S.; Cui, Y. Atomic structure of sensitive battery materials and interfaces revealed by cryo–electron microscopy. *Science* **2017**, *358*, 506–510, Publisher: American Association for the Advancement of Science.
